# A Neonatal Case With Perinatal Lethal Gaucher Disease Associated With Missense G234E and H413P Heterozygous Mutations

**DOI:** 10.3389/fped.2019.00201

**Published:** 2019-05-22

**Authors:** Meili Wei, Aiqin Han, Liping Wei, Liji Ma

**Affiliations:** ^1^Department of Pediatrics, Zibo Central Hospital, Shandong, China; ^2^Sixth People's Hospital of Zibo, Zibo, China

**Keywords:** hepatosplenomegaly, thrombocytopenia, convulsions, premature neonate, ichthyosis

## Abstract

Perinatal lethal Gaucher disease (PLGD), a particular and serious form of type 2 Gaucher disease (GD), often causes lethality *in utero* or death within hours after birth. The typical clinical manifestations include non-immune hydrops fetalis (NIHF), premature birth, fetal growth restriction, fetal intrauterine death, or neonatal distress and rapid death after birth. Here, we present a premature neonate with GD whose main clinical manifestations included intrauterine growth retardation, anasarca, facial dysmorphia, ichthyosis, respiratory distress, hepatosplenomegaly, joint contractures, myoclonus, refractory thrombocytopenia, anemia, elevated levels of liver enzymes, bile acid and direct bilirubin, cholestasis, pulmonary hypoplasia, intracranial hemorrhage, and abnormal electroencephalogram. The activity of β- glucocerebrosidase was 0 in the peripheral white blood cells of the neonate. The sequencing analysis identified the presence of missense G234E and H413P heterozygous mutations in glucerebrosidase (GBA) exon 7 and 10, with the latter first observed to be associated with PLGD. This infant died at 73 days of age.

## Introduction

Gaucher disease (GD) is the most common autosomal recessive inherited sphingolipidosis. Resulting from mutations in the gene of glucerebrosidase (GBA), an enzyme that hydrolyzes glucosylceramide into ceramide and glucose, the most typical clinical markers of GD is the significantly decreased *GBA* enzymatic activity (1). Until recently three types of GD have been recognized: type 1 (non-neuronopathic) is characterized by no neurological manifestations, type 2 (acute neuronopathic), and type 3 (subacute neuronopathic) are characterized by neurological impairment. Types of *GBA* mutations that cause the perinatal lethal Gaucher disease (PLGD) involve recombinant alleles, non-sense mutations, and missense mutations (2–9), of which homozygosity recombinant and fundamentally null *GBA* alleles cause the death of the fetus *in utero* or shortly after birth (2, 8). Diagnosis of PLGD is extremely difficult due to its rapid development which causes early lethality.

Here, we present a case of neonatal PLGD correlated to compound heterozygous G234E and H413P missense mutations in the *GBA* gene.

## Case Representation

### Presenting Concerns

**Figure 1:**
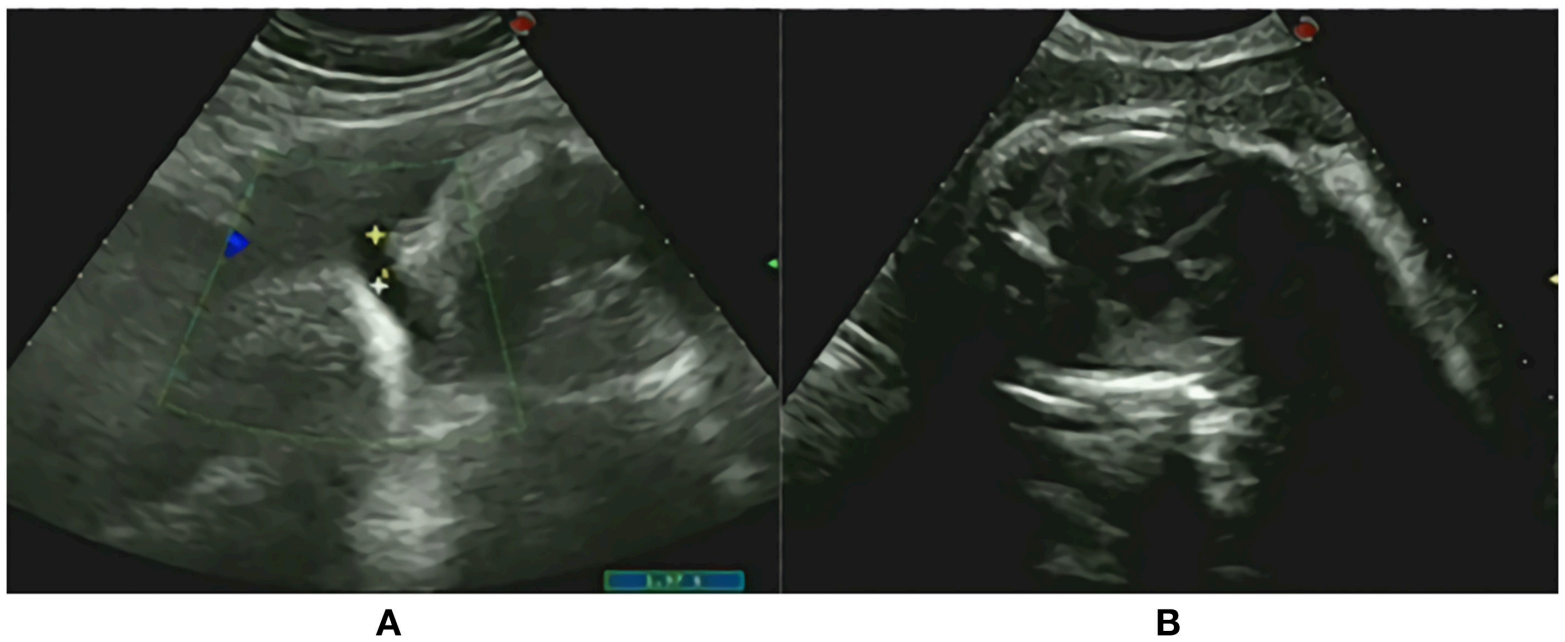
Prenatal onsets **(A)** oligohydramnios; **(B)** The normal heart silhouette, increased cardiothoracic ratio and small lung volume demonstrating pulmonary hypoplasia.

A male neonate weighing 2,020 g was given birth to by a 33-year-old Chinese mother (gravida 2, para 2) via cesarean section. The parents are non-consanguineous. During the first 5 weeks of pregnancy, the mother was treated with progesterone, and dydrogesterone until week 13 of gestation due to the signs of abortion. At week 24 of pregnancy, edema developed at the mother's lower limbs and gradually aggravated to anasarca at the end of the pregnancy. The mother was diagnosed with gestational diabetes at week 25 of gestation and her blood sugar levels fluctuated within a normal range through diet control. By 36 weeks, an ultrasound showed oligohydramnios and a normal heart silhouette, increased cardiothoracic ratio, and a small lung volume, indicating pulmonary hypoplasia (Figure 1). The family history was unremarkable.

### Clinical Findings

Labor was induced considering the oligohydramnios and a 3-h premature rupture of the fetal membrane at week 36. The placenta was grayish red and the focal purple red. No abnormalities were found in the umbilical cord. The Apgar scores of this neonate at 1 min and 5 min were 5 and 7, respectively. Ten minutes after birth, the neonate was admitted to the neonatal intensive care unit (NICU) for ventilation support due to tachypnea.

**Figure 2:**
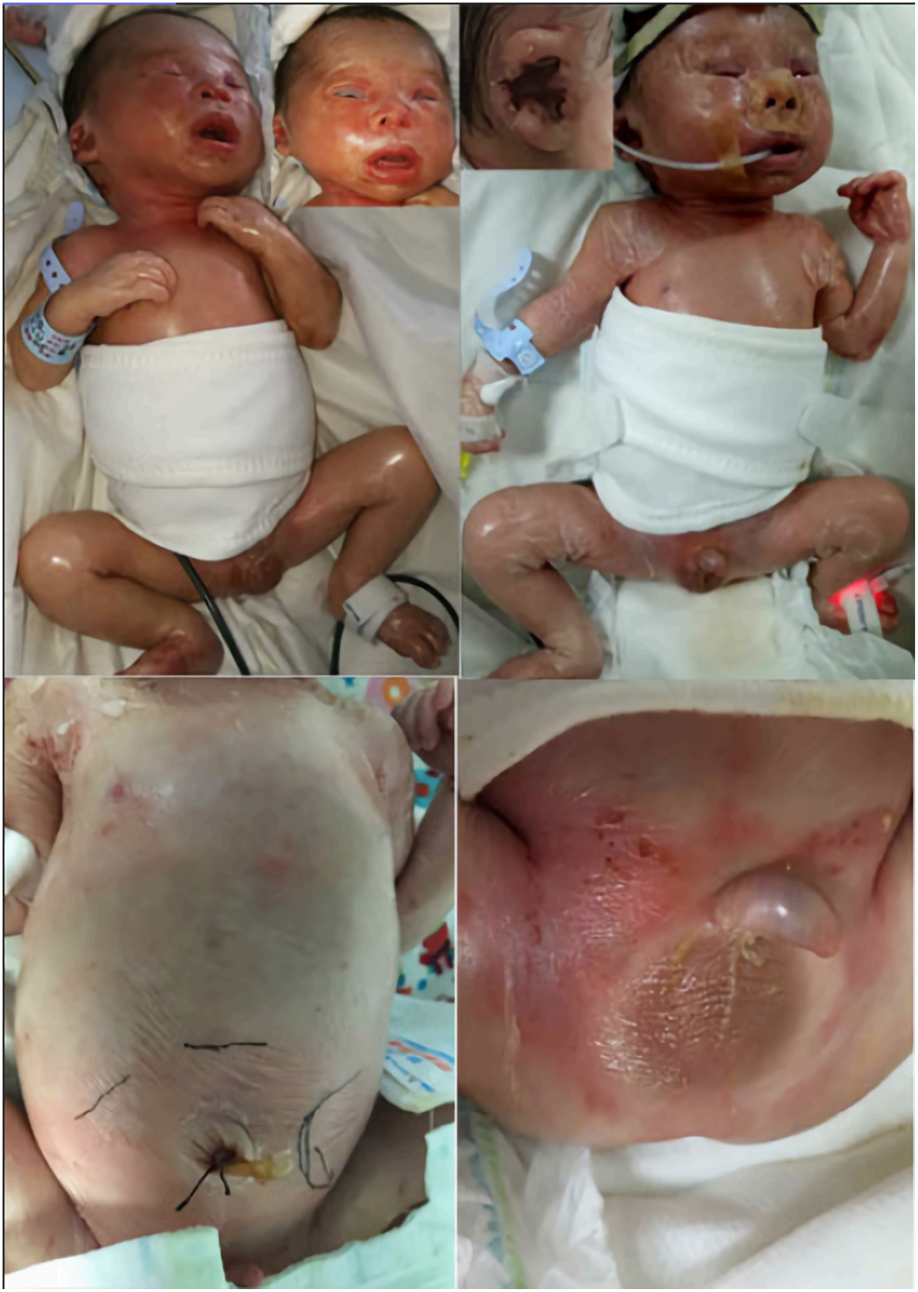
Clinical phenotype: facial dysmorphia; pinna development deformity; ichthyosis, joint contractures, anasarca; peeling skin on limbs and groin. The black line shows the boundaries of the liver and spleen, indicating hepatosplenomegaly; small microcaulia.

A physical examination on admission showed a weak response, weak crying, hoarseness, respiratory distress, positive signs of triple concave and anasarca. The neonate had many special clinical phenotypes (Figure 2). The facial features included dysmorphic hypertelorism, down-slanting eyes, an eye movement disorder, ectropion, hypophasis, thickening of the helix, constriction of the auricular rim, curl of the auricle and auricle cartilage, a flat nasal bridge, small nostrils, and everted lips. Ichthyotic and collodion skin covered the entire body with a shiny, red and tight appearance and was very friable, much like a layer of gelatinous film. The skin on the hands, feet, groin and scrotum was peeling extensively, and red tender skin was exposed. The breath sounds were clear, and no rales were heard during the auscultation of both lungs. A continuous machinery rumbling murmur was heard at the upper left sternal edge. A 3/6 pansystolic murmur was heard all over the precordium but clearest at the left sternal edge. The abdomen was markedly distensible. The liver was 5 and 6 cm under the right costal margin and under processus xiphoideus, respectively. The spleen was 6 cm below the left costal margin. Both organs had an extremely firm, stone-like texture when palpated. The neonate was immobile unless upon stimuli, accompanied by flexion contractures at the elbow and knee joint, hypertonia, akinesia. Myoclonic movements were noted soon after birth. Myoclonic seizures manifested through limb jitters that lasted about 10 s before alleviation. The bilateral testis did not descend to the scrotum. The neonate had small microcaulia. Primitive reflexes were not elicited.

### Diagnostic Focus and Assessment

At birth, the CBC indicated a platelet count of 19,000/mm^3^, WBC 11,880/mm^3^, and hemoglobin at 143 g/L. At 2 d of age, the CBC indicated the platelet count at 8,000/mm^3^, WBC 13,170/mm^3^, and hemoglobin at 165 g/L.

A liver function test noted hypoproteinemia (TP 53.1 g/L, ALB 26.9 g/L); elevated enzymes (AST 123.4 U/L); and cholestasis (TBILI/ DBILI 43/28 μmol/L, TBA 25 μmol/L, γ-GT 183U/L, ALP 249 U/L), LDH 326 U/L at birth. The peak values of TBILI 89 U/L, DBILI 69 U/L, TBA 37 μmol/L, γ-GT 773 U/L, ALP 296 U/L, LDH 1055 U/L.

The initial coagulation test showed the following: PT of 25.7 s, APTT of 40.4 s, TT of 26.4 s, INR of 2.08, FIB 0.79 g/L.

The neonate underwent a bone marrow puncture at 5 d of age because he had no response to intravenous platelet and gamma globulin treatment. Bone marrow aspiration indicated proliferous granulocytic cells and erythroid cells. The ratio of lymphocytes was high. Three megakaryocytes were found in the whole smear. No Gaucher cells were found.

The pathology of the placenta, fetal membrane and umbilical cord showed chronic inflammation with placental villi denatured necrotic calcification focus and umbilical cord arteriovenous congestion and focal hemorrhage.

At birth, an echocardiography revealed (1) normal size of the atrium and ventricular chamber, normal thickness of the ventricular wall, normal structure of the valve; (2) normal connection and inner diameter of the aorta and pulmonary artery; (3) LVEF 62%; (4) CDFI: a patent ductus arteriosus with continuous left-to-right shunt signal between the root of the left pulmonary artery and the initial segment of the descending aorta, a beam width of 1.5 mm; ventricular septal defect with small left-to-right shunt signal in the middle segment of the ventricular septum during systole, a beam width of 1.2 mm, Vmax 3.0 m/ s, PGmax 37 mmHg measured by CW; a moderate reflux signal at the atrial side of the tricuspid orifice during systole, Vmax 3.3 m/ s, Pgmax 44 mmHg, moderate tricuspid regurgitation measured by CW, the pulmonary artery systolic pressure of 54 mmHg estimated by the degree of tricuspid regurgitation.

**Figure 3:**
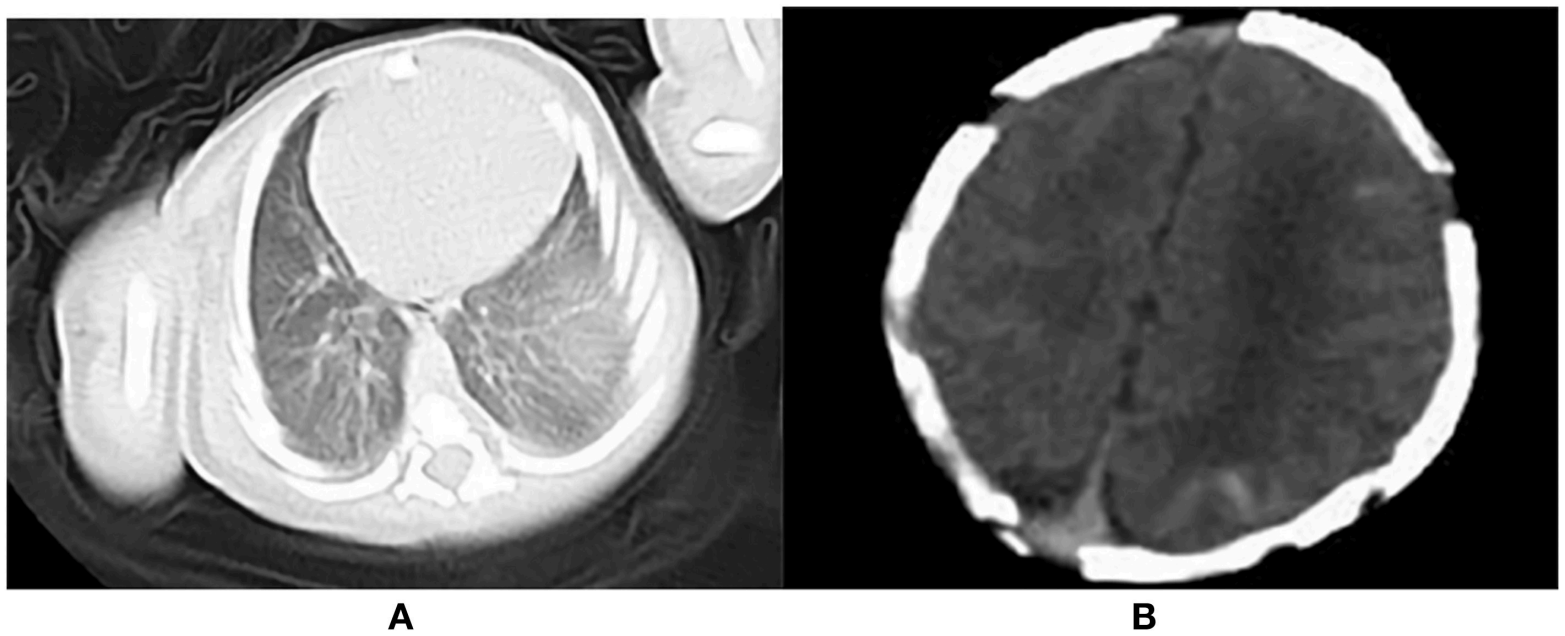
**(A)** Lack of air in the lung; **(B)** Multiple hemorrhagic lesions in the left frontal, temporal and parietal lobes.

An abdominal ultrasound showed palpable hepatosplenomegaly and cholestasis. A genital ultrasound revealed that the bilateral testis had hydrocele. At 2 d of age, lung computed tomography indicated a lack of air in the lungs (Figure 3A), and a brain computed tomography revealed multiple hemorrhagic lesions in the left frontal, temporal and parietal lobes (Figure 3B). At 9 d of age, a craniocerebral MRI disclosed a small hemorrhagic lesion in the left parietal lobe accompanied with a widening of part of the external space. At 14 d of age, the neonate developed frequent tonic seizures and an electroencephalogram showed sharp waves, spike waves, and outburst suppressions. The sleep cycle was not observed. At 16 d of age, no hemorrhagic lesions were found in the craniocerebral MRI.

At 19 d of age, an echocardiography demonstrated LVEF 63%, VSD with small left-to-right shunt signal and a pulmonary artery systolic pressure of 47 mmHg. At 35 d of age, an echocardiography demonstrated LVEF 60%, VSD and a pulmonary artery systolic pressure of 38 mmHg.

Cytogenetic evaluation revealed 46 XY. No copy number variation associated with clinical symptoms was detected in the whole genome copy number variation analysis.

We correlated all the aforementioned manifestations with a possible metabolic disease. At 44 d of age, the leukocyte enzyme assay for β-glucocerebrosidase revealed an activity of 0.00 nmol/h/mg (control range: 10–25 nmol/h/mg), explicitly supporting the diagnosis of PLGD. Testing of the mother and father revealed serum β-glucocerebrosidase enzymatic activities of 13.3 and 17.8 nmol/h/mg, respectively. Urinary screening for metabolic disorders by GCMC was negative.

**Figure 4:**
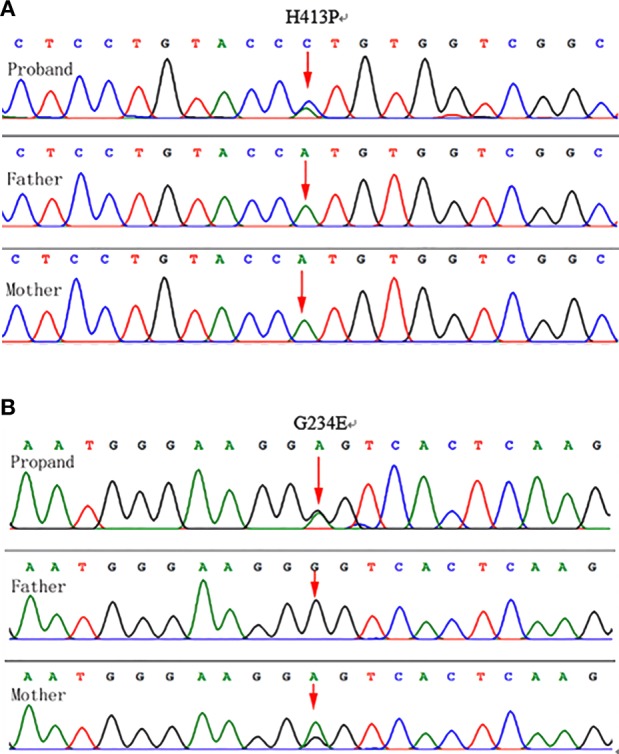
Genotyping results: **(A)** Chromatograms showing DNA sequencing results for the proband and parents. Genomic DNA isolated from leukocytes (proband, mother, and father) demonstrated a novel heterozygous A-to-C transition at position 1,238 in exon 10 of the GBA gene was identified in the proband, which resulted in a His413Pro mutation. The mutation was not detected in the parental genomic DNA. **(B)** A heterozygous G-to-A transition at position 701 in exon 7 of the GBA gene in the mother and the proband, which resulted in a Gly234Glu mutation in the maternal and proband's genomic DNA.

The proband's *GBA* sequencing analysis demonstrated two heterozygous mutations, Gly234Glu (c.701G>A; p.G234E) in exon 7and His413Pro (c.1238A>C; p.H413P) in exon 10 (Figure 4).

### Therapeutic Intervention

Following admission to the NICU, the patient was given non-invasive respiratory support. Without sucking and swallowing, he was always kept fed through a gastric tube. With refractory thrombocytopenia, he was treated with 2 days of an intravenous infusion of gamma globulin (1 g/kg/d) and intermittent multiple transfusion of platelets; the platelet count fluctuated between 22,000 and 68,000/mm^3^. After treatment with intravenous infusion of vitamin K, ethamsylate and cryoprecipitate, anomaly coagulation parameters were improved. Levetiracetam, and phenobarbital were administrated orally to control the severe myoclonus, but the symptoms were poorly controlled. At 44 d of age, with the confirmation of PLGD diagnosis, the treatment was discontinued upon the parent's request. The patient was then discharged and taken home.

### Follow-Up and Outcome

During the follow-up period, the patient kept gastric tube feeding, had no increase in physique, and gradually developed a pale face, obstinate hiccups, progressive neurological deterioration, hepatosplenomegaly, abdominal varicose veins, systemic edema (especially in the lower limbs and scrotum). He was supplied with humidified oxygen via a face mask, adequate caloric intake was ensured and excessive salt intake was avoided. He was also given dihydrochlorothiazide, spironolactone, and lanatoside C. However, respiratory failure, hepatic failure, and renal failure gradually occurred. The patient died at the age of 73 days.

## Discussion

The PLGD has a unique phenotype in addition to the classical type 2 GD symptoms (3).

The clinical manifestations of PLGD include NIHF, premature birth, fetal intrauterine death, or neonatal distress and rapid death after birth. Having reviewed published cases, Mignot et al. suggest (9) that the following significant prenatal manifestations of PLGD could be detected by ultrasonography: polyhydramnios, NIHF, fetal growth restriction, hepatosplenomegaly, acinesia, joint contractures, cerebral ventricular dilatation, and small bowel hyperechogenicity. The significant postnatal signs of PLGD include hepatosplenomegaly, akinesia and arthrogryposis, non-specific facial dysmorphia, collodion aspects of the skin, jaundice, severe thrombocytopenia, purpura, anemia, clotting factors deficiency, hemorrhages, and the elevation of liver enzymes. Frosk et al. reported a case of proven PLGD with cerebellar hypoplasia and pulmonary hypoplasia (10).

The neurological involvement of PLGD is similar to that of classical type 2 GD, but

with rapid aggravation. Histological analysis of autopsy specimens reveal severe apoptosis (11, 12), indicating *in utero* central nervous system damage. The severity of neurological impairment was closely associated with neurodegeneration (12).

The common non-neurological features of GD are diversely encountered in PLGD. However, ichthyosiform skin abnormalities and unusual craniofacial dysmorphic features, such as microcephaly, a flat nasal bridge, reversed lips and microstomia were described (13). Dysmorphic features are observed in about 30% of PLGD infants (14). The association between congenital ichthyosis and PLGD was firstly noted in 1988 in two Lebanese siblings with the collodion baby phenotype (15). Many case reports indicate that congenital ichthyosis is part of the PLGD spectrum (16). Congenital ichthyosis occurs in the whole body, predominately on the palms and soles or in flexure folds (17).

The retrospective comparison presents a similarity between severe multisystem involvement observed in our patient and symptoms reported in the above literature. Of all the clinical signs, what prompted the laboratory investigation for GD was hepatosplenomegaly, thrombocytopenia, ichthyosis, and facial dysmorphia.

In this study, two complex heterozygous mutations G234E and H413P were detected in the proband. The mutation G234E (c.701G>A) from the maternal source has been reported in patients with GD. The mutation H413P (c.1238A>C) was unique in the proband's DNA, it was a spontaneous novel mutation that had, to our knowledge, never been reported in GD patients. No previous correlation between the heterozygous mutation H413P (c.1238A>C) and GD was found. This locus had no frequency in the gnomAD database and was excluded as a polymorphic locus. The locus was predicted to be a harmful mutation by the SIFT and PolyPhen2 protein functional online analysis software. MutationTasler also predicted it pathogenicity. The effects of two heterozygous mutation sites on the spatial structure of the *GBA* gene were analyzed by SWISS-MODEL software. After the substitution of the glycine at position 234 by glutamate of the *GBA* gene, the hydrogen bond broke between position 232 and position 234. A hydrogen bond formed between position 412 tyrosine and position 414 valine of *GBA* gene, after the substitution of the histidine at position 413 by proline. β-glucocerebrosidase activity of the mother with G234E heterozygosity was normal. However, the addition of a new mutation H413P caused a complete loss of enzymatic activity in our patient. We hereby postulate that the H413P mutation, a mutation that possibly alters the structural properties at the active site and hence fundamentally eliminates the enzymatic function, may be responsible for the early lethality of patients with PLGD. It may be a serious and harmful mutation.

In summary, we describe the clinical and molecular findings in a Chinese neonate with perinatal lethal phenotype of GD. Clinical manifestations such as NIHF, hepatosplenomegaly, arthrogryposis, and facial dysmorphism should raise suspicion of PLGD. In addition, genetic counseling and patient family management both contribute to an accurate and timely diagnosis of GD. Experience from this case may help determine the protocol of a rapid diagnosis of PLGD.

## Ethics Statement

This study was carried out in accordance with the Ethical Review Committee of the Zibo Central Hospital. Written and informed parental consent was obtained for publication of this case report.

## Author Contributions

MW designed the study, collected data, drafted the initial manuscript and carried out the initial analyses. LM reviewed and revised the manuscript. AH and LW collected collected and collated clinical data of the patient.

### Conflict of Interest Statement

The authors declare that the research was conducted in the absence of any commercial or financial relationships that could be construed as a potential conflict of interest.
